# A Comprehensive WSN-Based Approach to Efficiently Manage a Smart Grid

**DOI:** 10.3390/s141018748

**Published:** 2014-10-10

**Authors:** Ruben Martinez-Sandoval, Antonio-Javier Garcia-Sanchez, Felipe Garcia-Sanchez, Joan Garcia-Haro, David Flynn

**Affiliations:** 1 Department of Information and Communication Technologies, Universidad Politécnica de Cartagena (UPCT), Campus Muralla del Mar, E-30202 Cartagena, Spain; E-Mails: rms3@alu.upct.es (R.M.-S.); felipe.garcia@upct.es (F.G.-S.); joang.haro@upct.es (J.G.-H.); 2 School of Engineering & Physical Sciences Mountbatten Building Edinburgh, Heriot-Watt University, Edinburgh, EH14 4AS Scotland, UK; E-Mail: d.flynn@hw.ac.uk

**Keywords:** smart grid, wireless sensor network, power asset, IEEE 802.15.4, MicaZ

## Abstract

The Smart Grid (SG) is conceived as the evolution of the current electrical grid representing a big leap in terms of efficiency, reliability and flexibility compared to today's electrical network. To achieve this goal, the Wireless Sensor Networks (WSNs) are considered by the scientific/engineering community to be one of the most suitable technologies to apply SG technology to due to their low-cost, collaborative and long-standing nature. However, the SG has posed significant challenges to utility operators—mainly very harsh radio propagation conditions and the lack of appropriate systems to empower WSN devices—making most of the commercial widespread solutions inadequate. In this context, and as a main contribution, we have designed a comprehensive ad-hoc WSN-based solution for the Smart Grid (SENSED-SG) that focuses on specific implementations of the MAC, the network and the application layers to attain maximum performance and to successfully deal with any arising hurdles. Our approach has been exhaustively evaluated by computer simulations and mathematical analysis, as well as validation within real test-beds deployed in controlled environments. In particular, these test-beds cover two of the main scenarios found in a SG; on one hand, an indoor electrical substation environment, implemented in a High Voltage AC/DC laboratory, and, on the other hand, an outdoor case, deployed in the Transmission and Distribution segment of a power grid. The results obtained show that SENSED-SG performs better and is more suitable for the Smart Grid than the popular ZigBee WSN approach.

## 1. Introduction

The Energy Information Administration (EIA) [1] has recently forecasted that world energy consumption will grow by 56% between 2010 and 2040. Although 80% of this energy is derived from traditional fossil fuels, renewable energy and nuclear power are the world's fastest-growing energy supplies—each with an increase of 2.5% per year. The increase in the number of different energy sources the current power grid has to accommodate and regulate leads to an unavoidable increase in the complexity of grid; despite that, this power grid still lacks an effective sensing and control platform that could help provide more intelligence to the management process. To alleviate this situation, cutting-edge sensing and control technologies are envisioned to be integrated into the current power grid. This integration will result in what is called the Smart Grid (SG), a more configurable, dynamic, reliable, flexible and effective power network.

The Smart Grid can be observed as the evolution of the primitive state of the current electrical grids which in most cases consist of power transmission lines more than 50–60 years old [2] and whose conceptual design has remained unchanged for more than 100 years [3]. This out-of-date design, together with the lack of information collecting mechanisms, have been identified as the main triggers for serious power failures such as the European blackout on November 4, 2006 that affected more than 15 million European household) [4] and the North American black out in 2003, with an estimated cost of four to 10 billion dollars to the U.S. economy [5]. In light of this situation, it should be one of the priorities of every country's economy to provide their power grids with the required tools to enable intelligent asset management and successful health management. In this regard, successfully managing key power assets (substations, transformers and transmission towers among others) is considered to be as important as being able to predict their remaining useful life (RUL) or the time to the next failure. For such purposes, relevant data must flow in both directions: from power assets to central processing centers (sending acquired physical data such as temperature, voltage, vibrations, *etc.*) and from there to power assets (making decisions such as for instance, triggering the ventilation system when the temperature increases in an indoor substation or balancing the incoming voltage) [6,7].

Wireless Sensor Networks (WSNs) are considered to play an important role in this two-way communication process, which is in charge of bringing intelligence to the electrical network. This enabling technology has proven to have the ability to promote the use of automation, control and sensing techniques at a low cost and with very low-power consumption. Although other alternatives like wired communication systems could be considered as a more robust solution, they would require much more investment (very high deployment costs), an increase in maintenance costs and less network scalability, therefore leading to an inflexible communication solution for the new grid [8]—the network would not be able to accommodate to new requirements. The inherent decentralized nature of wireless systems, the decreasing evolution of prices, their easy and fast plug-and-play philosophy and a long-term durability have made WSN technology a clear winner in the field of power engineering [9].

The substantial benefit of a WSN lies in its capacity to deploy small battery-powered sensor nodes directly into critical power assets with practically no installation and maintenance work. These devices wirelessly send their processed sensed data to central nodes (also known as sink nodes) where data is converted into decisions and sent back to nodes. Critical power assets can be found along the three distinct segments of the electrical grid: Generation segment (where power plants and energy storage systems can be found), Transmission and Distribution (T&D) segment (where transformers, substations and power lines are installed) and Consumers segment (where end-users' premises are).

However, the concern behind this approach is that designing the required WSN-based monitoring systems implies a profound knowledge of telecommunications and electronics involved, especially when dealing with an integral *ad*-*hoc* solution. Furthermore, because of the particular nature of the power grid (mainly very harsh propagation conditions and the lack of appropriate inexhaustible power sources), systems intended to manage and control power assets have to fulfill specific requirements and be engineered with those requirements in mind. Therefore, the communication protocol stack and technologies used in WSNs for the Smart Grid must be revised and improved (when not specifically designed from scratch) to achieve the best results. Unfortunately, to the best of our knowledge, no integral *ad*-*hoc* WSN solution has been proposed in the Smart Grid research field so far and only few approaches have intended to adapt well-known WSN technologies originally thought to operate in different fields [10–13].

To address this shortcoming, and as the main contribution to this work, we propose a fully integral *ad*-*hoc* control and sensing solution based on wireless sensing technologies (denoted as SENSED-SG) satisfying the major requirements of a Smart Grid monitoring application. This control and sensing solution is achieved by means of a novel protocol stack designed from scratch to appropriately exploit the benefits the SG and circumvent potential challenges. Special attention has been paid to the MAC and Network layer where the core of the communication system lies and its main settings tuned to attain good performance. In addition, our proposal has been evaluated and validated by means of theoretical analysis, computer simulation and extensive experimentation under controlled conditions: (i) a High Voltage AC/DC laboratory and (ii) the T&D segment of a power grid. As an additional contribution, our solution is compared to ZigBee, one of the most popular standards in WSNs in a Smart Grid environment. As far as we know, this is the first test-bed including multi-hop and cluster-tree topologies performed in a Smart Grid environment. The results reveal that our *ad*-*hoc* solution, specifically designed for the Smart Grid area, is better suited than other solutions (like ZigBee) adapted to operate in this research field.

The rest of this paper is structured as follows. Section 2 presents an up-to-date review of the particular requirements of a Smart Grid and the challenges the enabling technologies have to face. Section 3 introduces the related work found in the literature, summarizing their advantages and limitations. Section 4 describes our proposal as well as the mathematical analysis supporting the decisions made. The set of complex simulations conducted is reported in Section 5. The test-beds accomplished are explained in Section 6. Finally, Section 7 concludes and outlines future works.

## 2. Requirements and Challenges of the Smart Grid

The first step in the design of a sensing and control system based on a WSN solution must be the definition of the requirements that the system must satisfy. Generally speaking, the main goal of the Smart Grid is to improve the efficiency, reliability and safety of the current power grid while easing the process of integrating new sources of energy (e.g., renewables). This is accomplished by the inclusion of control, monitoring and processing tools into the current power grid. These three tools can be effectively implemented with the deployment of a ubiquitous WSN underpinned by a real-time processing support system. WSNs, like any other technology, have to face diverse challenges to achieve their full effectiveness, especially when dealing with noisy and unsteady environments.

It is a common practice in the scientific literature to describe the requirements that an ideal Smart Grid must fulfill without taking into account the limitations of the enabling technologies [14,15]. This leads to a deficient understanding of the underlying mechanisms that make a Smart Grid possible and may cause an incorrect vision of the whole picture. To this aim, the following sub-sections summarize the primary requirements of the Smart Grid and connect them with the potential challenges that WSN need to tackle satisfactorily.

### 2.1. Scalability

The sensing and control system must work in optimal conditions even when the Smart Grid grows significantly. A typical utility of 25,000 km of high voltage power lines and thousands of capacitors and transformers could require the monitoring of over 100,000 distinct elements and distributed sensors or sources of data that may be spread over a 20–80,000 sq. km area [16].

From the technologies' perspective, there are a couple of challenges to be faced in order to provide high scalability to a Smart Grid. First of all, the information network must be able to grow inexpensively and on demand, to enable this, monitoring systems composed of WSN devices must be of very low cost and be available to designers and end users. Secondly, when the network increases in terms of WSN devices it does also in size. It is therefore of paramount importance the implementation of multi-hop algorithms allowing the network to operate for long distances and providing communication from any arbitrary element of the Smart Grid to the central node [17,18].

### 2.2. Long-Standing Platform

Power assets must be designed and engineered to ensure a proper operation during a considerable amount of time—most of the current American power grid is about 50–60 years old [16]—therefore, in a Smart Grid, any monitoring and actuating system must be ready to work with minimum maintenance for extremely long periods of time.

WSNs are composed of a large number of hardware-constrained sensing nodes whose operation periods must be adjusted to mitigate the lack of available, inexhaustible, power supplies and guarantee an appropriate lifespan. In this regard, it is worth remarking that there is no segment of the power grid where voltages are suitable for supplying energy to sensor nodes (working at 5 V), and, as a solution, voluminous and expensive voltage transformers should be deployed for every single node in order to connect them directly to the power grid. This concern is especially important in the case of the T&D segment where voltages are usually greater than two hundred thousand volts and the installation costs to power WSN nodes via traditional electric wiring would be unaffordable. On the other hand, energy-harvesting techniques (solar, vibrations, electromagnetic field, wind, *etc.*) are a promising research topic for empowering small and isolated WSN devices in the Smart Grid. The goodness of this approach is heightened by the high electromagnetic fields found in power grid environments and many works have already corroborated the efficacy of it in a Smart Grid context [19,20]. However, solar energy is not a feasible approach for indoor environments and not reliable in many countries where the solar energy harvested may be simply insufficient during certain seasons. On the other hand, and to the best of our knowledge, there is no current off-the-shelf sensing device fitted with the other aforementioned energy harvesting mechanisms (electromagnetic fields, vibrations, wind, *etc.*) that has already been tested in the Smart Grid. Therefore, the inclusion of these mechanisms is not covered in this work—although it is considered as a future potential improvement.

### 2.3. Reliability, Robustness and Noise Resilience

Electronic devices may become inoperative or fail because of the presence of very high electromagnetic disturbances nearby as explored in [21]. As a result, a malfunctioning power asset can be not identified as such, provoking partial (isolated) outage or overall system failures which can affect larger regions and a higher number of consumers due to the cascading effect [22]—as it happened in the aforementioned European blackout. Therefore, the endurance of the WSN devices and a prompt detection of the failing ones is a must to prevent malfunctioning power assets from impoverishing the capacity of the power network. In this regard and from a global perspective, the correct operation of the whole information network must be guaranteed during the entire lifespan, providing specific mechanisms to resist and/or recover from harsh propagation conditions, severe weather conditions, strong electromagnetic interferences, equipment degradation, *etc*.

From a technological point of view, it is a common denominator of any power network the presence of high electromagnetic disturbances which strongly deteriorate the performance of any wireless network and, in particular, of a WSN deployment. These high electromagnetic disturbances along with the absence of unlimited power sources are two of the most important concerns for a WSN operating in a power grid environment [21,23]. Our SENSED-SG is engineered having taken these effects into account, thus offering better network performance in comparison with other wireless technologies (e.g., Wi-Fi or Wi-MAX).

The harsh propagation conditions found in power grid environments are mainly caused by:

- Electromagnetic interferences caused by current lines and power assets in general.
- Multiple metallic structures that may reflect and distort waves and cause noise-cancellation phenomena.
- Multiple objects blocking line of sight.
- Other communication systems working in the same frequency such as Wi-Fi, Bluetooth, Cordless Phones, *etc.* (mainly in substations found in industrial areas).

Although many current systems have some mechanisms to deal with these phenomena (DSSS techniques, MIMO-antennas, *etc.*), further efforts are needed to improve communication quality in such harsh environments. SENSED-SG makes use of many of the well-known techniques (noise-tolerant modulation techniques, timed-out retransmissions, use of the least-utilized frequency channel, *etc.*) and implement other novel solutions as explored in the following sections.

Another aspect to consider is the failure of a particular WSN node. Under these circumstances, the network topology may change causing neighboring nodes of the faulty node to seek alternative routes to the central station. Consequently, the routing protocol must provide explicit mechanisms to refresh and discard any possible corrupted route.

### 2.4. Security Issues

According to the Electric Power Research Institute (EPRI), one of the emergent requirements facing the Smart Grid development is related to cyber security of systems [15]. The growing importance of the Smart Grid make it a sensitive target for cyber terrorists, which implies a critical concern for system designers as remarked in the EPRI report [24]. Therefore, it is mandatory that the information collected and the decisions made are sent encrypted to prevent malicious users from eavesdropping or tampering with sensitive information as many works have already outlined [25,26].

For small embedded devices like WSN nodes, implementing state-of-the-art encryption techniques represents an important challenge due to their hardware constraints, both in terms of computing capabilities and memory. Besides, the required extra computation leads to a non-trivial increase in the power consumption. Therefore, a thorough study of the current available encryption algorithms to select the best solution is needed. However, the National Institute for Standards and Technology (NIST) and the Federal Information Processing Standard (FIPS) has determined that the preferred solution for new deployments should be AES (Advanced Encryption Standard) to guarantee the required level of privacy in Smart Grid environments [15].

### 2.5. Real-Time Monitoring

The network must acquire, process and analyze data from the power assets in a suitable period of time to allow decision-making and control algorithms running in the central node to react against these changes appropriately. In this regard the differences between the asset management and the power system protection should be noted. While the former does not impose hard constraints in terms of real-time processing and generates large amount of data, the latter inherently requires a strict real-time decision-making system and dispatches fewer packets [21,27]. It is accepted by the research community that measured data in wireless asset-managing systems, like the one proposed here, should reach sink nodes in less than 15 s [15]. It is important to highlight, that such constraint only applies to wireless asset-managing system and other systems (wired or those intended to be used in power system protection) may have to meet other requirements (such as those suggested in the IEC61850 standard).

From a technological point of view, to ensure an appropriate asset managing solution in terms of timing constraints, diverse aspects must be taken into account. Firstly, despite the WSN nodes of the Smart Grid are able to collect large amounts of data, this information has to be pre-processed in every node before being sent to the wireless network. This is carried out to prevent large volumes of data from simultaneously reaching the central node, negatively affecting its ability to furnish a real-time decision making system. It has been proven as a non-trivial issue in widespread WSN sensing systems such as the London Traffic Management system [28]. Secondly, WSN nodes are extremely hardware limited devices incapable of computing complex mathematical operations, therefore there must exist a study of the trade-off between pre-processing in the node and the number of packets dispatched to the network.

Moreover, to guarantee adequate data collection times, data latency must rank within a tolerable range. To achieve this goal, specific protocols settings must be tweaked as will be explored later in Section 5.1. Finally, extensive tests must be carried out to validate the deployed solution in terms of end-to-end delay and to ensure the proper functioning of the network.

### 2.6. Interoperability

In the framework of a Smart Grid, interoperability means the ability of diverse systems to work together, exchange information or equipment from each other and operate cooperatively to perform several tasks [15]. To provide a suitable level of interoperability and a seamless data flow among WSN devices, the use of standards is a key issue [29,30].

To accomplish this, the hardware platform and software architecture of the implemented solution must be well known by the research/engineering community. Furthermore, the communication protocols and message exchange patterns must be fully documented to facilitate interoperability with other potential systems.

## 3. Related Work

Many papers have looked into the requirements of a Smart Grid as the main way to understand the big picture [14,15,27], on the other hand many others have kept the focus on the challenges diverse technologies have to face in order to implement a Smart Grid [16,31]. However, as far as we know, there is no single work that unifies these two aspects—requirements of a Smart Grid and what challenges the enabling technologies have to tackle to achieve them—with the aim of setting the starting point for a Smart Grid suitable control and sensing solution.

Works in [3,32,33] have studied the performance of some technologies/protocols and their behavior in “simulated” smart-grid environments obtaining good results. Nevertheless, we believe that individual technologies that are interconnected to form a bigger system cannot be analyzed independently since doing so will overlook the non-trivial interaction between them. For instance, in [34] a new secure routing protocol including quality of service support for Smart Grids is presented and analyzed. Although several simulations are shown and their outcomes discussed, the authors omit the effect of an underlying MAC (Medium Access Control) layer that may severely deteriorate the results (e.g., it is notorious that many MAC protocols do not appropriately deal with certain types of data traffic like broadcasts or multicast packets). In this regard, our analyses and test-beds are aimed at providing results of different performance metrics considering every possible interaction between protocols—as will be reflected in Sections 6 and 7. In short, a complex system cannot be analyzed as the sum of its composing elements.

In [35], a performance evaluation of a ZigBee-based approach for different Smart Grid environments is carried out by means of ns-2 simulations. However, the paper depicts an unrealistic scenario for a Smart Grid since every sensor node has a direct connection (no intermediate hops) with its sink; thus, leaving out the critical impact on the performance of a routing algorithm. Regarding ZigBee standard, many works have studied the suitability of this solution for the Smart Grid field [9,10,36], but ignored in which segment of the power distribution grid the standard would be deployed. This is an important fact in multi-hop networks, since ZigBee requires the presence of router devices for the network to be set up, and these devices entail a higher consumption of energy, as will be explained in the following sections. This situation forces devices to be connected to a power source to ensure a long lifespan; thus, clearly restricting the applicability of such networks to residential zones where nodes can be more easily powered. Therefore, our work pays particular attention to the segment of Transmission and Distribution since, in our opinion, this is the segment where current solutions are less effective.

Other potential technologies like PLC (Power Line Communications) and Wi-MAX have been studied in [15,27,31] and their advantages outlined. However, it must be understood that a network based on PLC would have two major drawbacks: firstly, if there exists a breakdown in the actual electricity line, communications would be impossible. Secondly, the scalability of the solution is quite restricted due to the short bandwidth that this technology provides [37]. On the other hand Wi-MAX is only suitable for gateways or sink nodes (connected to power sources) because of its very high power consumption.

## 4. SENSED-SG Design and Protocols

Our WSN-based solution called SENSED-SG furnishes a comprehensive asset management system for the Smart Grid that seeks from its very beginning to meet every imposed requirement and circumvent all the technological challenges. For the sake of clarity and due to the limitations of current solutions (e.g., ZigBee), our analyses and studies are focused on the T&D segment without any loss of generality. Going into detail, we implement specific communication layers to satisfy the main requirements mentioned above.

The WSN here proposed is composed of COTS (Commercial Off-The-Self) devices (*i.e.*, MicaZ [38]) as a platform for sensor integration. This choice is motivated by the following reasons. Firstly, the use of well-tested open-hardware and low-cost COTS sensing devices improves the reliability of our solution and eases future potential hardware replacements. Secondly, MicaZ-compatible devices show a very low power consumption, even when compared with other low-power consumption alternatives as demonstrated in [39] and [40]. To help further reduce power consumption and boost network performance, MicaZ integrates in its platform the CC2420 radio interface that has been demonstrated to be reliable and effective enough even under harsh interferences due to the adoption of DSSS and OQPSK modulation techniques [41]. And lastly, the TinyOs operating system [42] is fully supported by this platform. It is the preferred operating system for many wireless sensor-based systems [43–45], Moreover, many studies show its good performance (in terms of processing and memory resources) and energetic efficiency in comparison with other similar alternatives [46–48]. Next subsections explain and justify our proposal from an architecture protocol perspective and following a bottom-up approach.

### 4.1. Physical Layer

There is no doubt that the release of the IEEE 802.15.4 standard for the physical and medium-access layers have paved the way for the expansion of WSN technology in a wide variety of productive fields [49]. This standard has become the de-facto starting point for any low-data-rate low-power consumption wireless system that strives for interoperability. The IEEE 802.15.4 supports three frequency bands, namely: 868 MHz, 915 MHz and 2450 MHz. However, the 2450MHz band is by far the most widely used because of (i) its higher data-rate (250 Kbps) compared to the other bands (20/40 Kbps respectively) and (ii) its noise-resilient modulation DSSS and O-QPSK [42]. Therefore, it is essential to ensure the adoption of this standard without any additional modification that could leave room for interoperability concerns.

### 4.2. SENSED-SG MAC Layer

**Figure 1.**
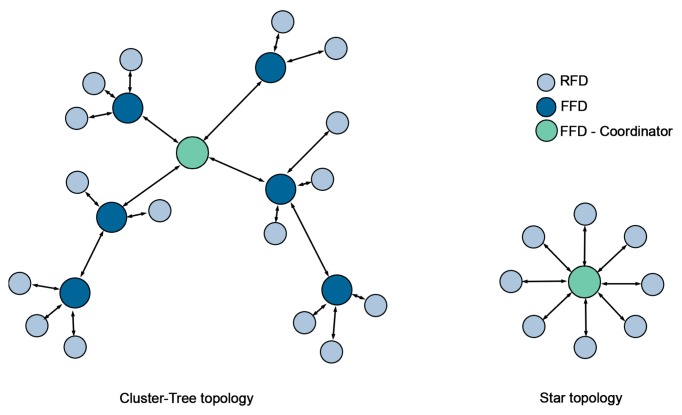
Cluster-Tree and Star topologies.

**Figure 2.**
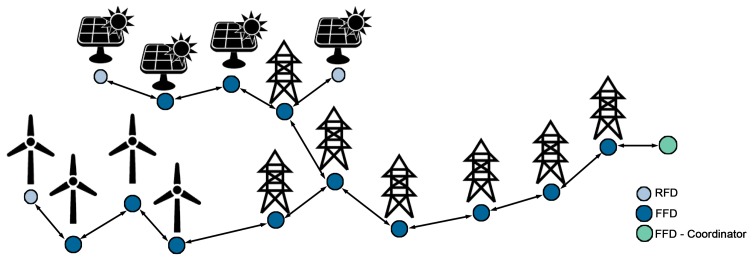
Growing pattern of a power grid.

Although the standard IEEE 802.15.4 MAC layer defines two different types of devices, full-function devices (FFD) –capable of routing– and reduced-function devices (RFD) –with no routing capabilities–, SENSED-SG only employs full-function devices. The use of both, FFD and RFD is based on the idea of implementing star or cluster-tree topologies (depicted in Figure 1) where reduced-function devices will always have full-function devices within their coverage to route their packets. The problem that arises with this solution is illustrated in Figure 2; when the area to cover grows in just one axis, which is quite usual in power grids and especially in the T&D segment, the number of FFDs increases sharply in relation to the RFDs. The result is a WSN basically composed of full-function devices, which (according to the IEEE 802.15.4 standard) either cannot enter into sleep mode (non-beacon-based networks) or must carry out laborious coordination tasks to enable a global synchronization of the network (beacon-enabled networks). Both strands lead to a significant reduction in network lifetime occurring, for instance, in ZigBee-based solutions. To mitigate this problem, we have designed and implemented a novel duty-cycling-based MAC layer focused on Smart Grid environments where full-function devices are capable of entering into sleep mode while still providing synchronization between peers (and, therefore, FFDs are not compelled to perform global synchronization tasks)—as explained hereafter in this section.

As above mentioned, the IEEE 802.15.4 standard proposes two different operational modes namely, beacon-enabled and non-beacon-enabled. In the latter, devices simply transmit using un-slotted CSMA-CA channel access mechanism—the medium is sensed and if detected free, the message is sent; otherwise (the medium is busy) devices delay the transmission a random period of time. In contrast, the beacon-enabled mode requires FFD coordinator (PAN -Personal Area Network- coordinator) nodes to periodically send beacons (control frames that inform other devices in their coverage range about the beginning of a period for sending their messages). This mechanism is commonly used in star topologies where there is always a coordinator device in coverage range. Nonetheless, although the standard is rather ambiguous in this aspect, beacon-enabled mode could also be implemented in cluster-tree topologies by means of tight global synchronization techniques. These techniques force every FFD to arrange its own beacon frames and unavoidably lead to an increase in code complexity and a sharp reduction in network scalability [50]—beacons must be coordinated in order not to collide with one another, which in turn, leads to a limit on the number of FFDs in a coverage area. Finally, note that beacon-enabled mode implies faster battery depletion in FFDs since every node involved in coordination tasks is required to continuously send beacon messages to let other nodes synchronize with it.

Therefore, in the interest of extending nodes' battery lifetime and increasing flexibility of the network topology, the non-beacon operational mode constitutes a cornerstone of our solution. Besides, the limit imposed in beacon-enabled networks on the number of FFDs coexisting in the same coverage area is especially important in Smart Grid environments and has been another reason for choosing non-beacon approaches—some segments of the power grid (like substations) are characterized for having a big number of power assets deployed in very small areas that can be potentially monitored; this greatly increases node density and thus potential beacon collisions. In addition, non-beacon mode simplifies the use of duty-cycle techniques that highly enhance power savings. The concept of duty cycle is based on the differences in power consumption of the radio module during the *awake period (ON)* and *sleep period (OFF)*. The ON period consumption (in which the radio is either passively or actively trying to receive messages) can be up to three orders of magnitude bigger than the OFF period (when the transceiver is off) [51]. Because of that, nodes will try to remain as much as possible in the former to extend their lifetime without neglecting their functionalities such as routing tasks, or data processing.

Concerning duty-cycling techniques for WSNs, WiseMac [52] and B-MAC [53] were the first protocols implementing such techniques denoted as Low-Power listening (LPL) in the MAC layer [54]. LPL proved its efficiency for WSNs by significantly reducing the power consumption of receiving nodes from nearly 50 mW to just 3.5 mW [53]. In particular, in B-MAC, a node willing to send a message would initiate its dispatching phase by broadcasting a void preamble signal which is long enough to guarantee all receiver nodes wake up (enter in their ON phase), sense the preamble, get ready (leave the radio ON) and wait for the reception of the message. The preamble acts as a “stay awake” signal; therefore, if a node wakes up, and after sensing the physical channel does not detect any preamble, it would immediately go back to sleep mode, thus saving battery. This solution, while simple, presents many drawbacks that were coped with with later protocols. In this sense, X-MAC [55] replaces the void preambles with a “packetized preamble” that identifies the recipient node of a specific message. This way, if a node wakes up and detects a preamble addressed to another node, it would directly go back to sleep mode. Furthermore, X-MAC includes the possibility for the receiver node to acknowledge the reception of a preamble by means of an ACK packet. In particular, when a sender node receives an ACK, it stops dispatching packetized preambles and directly sends its message. The objective is to reduce the number of preambles sent and, therefore, to extend the entire network lifetime. BoX-MAC-2 [56] takes as its starting point the X-MAC and B-MAC protocols but incorporates a significant feature: instead of sending preambles, BoX-MAC-2 dispatches the message several times as a preamble. This improvement greatly contributes to further reduce the power consumption in nodes by eliminating extra messages like the preambles and acknowledgements (in comparison with X-MAC). It is worth remarking that, by default, TinyOS makes use of the BoX-MAC-2 algorithm for its transmissions, and although having been proved as an acceptable solution, it leaves room for improvement.

Our solution here presented relies on the principles of the Contikimac [57] radio duty-cycling mechanism. It assumes the BoX-MAC-2 basis and, as an additional aspect, it includes a thin sender-receiver synchronization mechanism. This synchronization was firstly explored by A. El-Hoiydi and J.-D. Decotignie in their proposal for the Wise-MAC [52] algorithm, obtaining a notable savings of energy and an appreciable increase in the bandwidth utilization for WSNs. Instead of involving complex and tight clock-synchronization procedures (as in beacon-enabled ZigBee networks), this technique relies on a simple phase-lock mechanism: senders must learn when the receivers' radio is active (the ON phase of their duty cycle) so as to dispatch their packets at that precise moment. The phase-lock mechanism prevents senders from transmitting messages when receivers are not listening (their radios are OFF) which negatively affects their power consumption. Furthermore, since sender nodes will only use the wireless channel during short periods of time, other nodes will usually find it free, thus increasing the bandwidth utilization.

**Figure 3.**
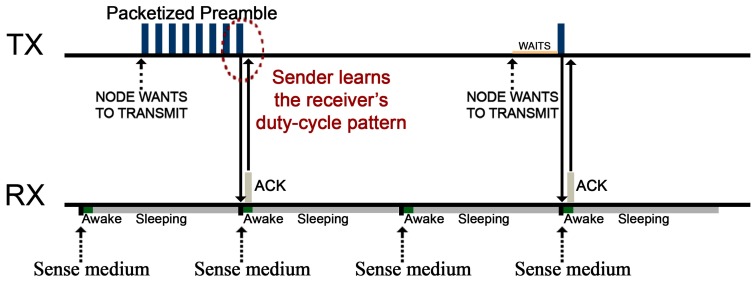
Contikimac.

The way senders learn receivers' duty-cycling phases is by means of link layer acknowledgements. The first time a sender wants to transmit to a specific receiver, it will continuously transmit its message (as if it were a preamble) until the receiver node receives such packet and acknowledges it by an ACK. When this occurs, the sender assumes the receiver is in the ON phase of its duty cycle—otherwise the receiver would not have heard and acknowledged the message. From that moment on, the sender will be able to predict when the receiver is going to enter in its ON phase before dispatching any message to it. It is worth noting that the duty-cycling mechanism only affects the radio receiving patterns, *i.e.*, a node can issue a transmission during its OFF phase if required by turning the radio ON previously and without affecting its duty cycle. The reason for this is that decoupling the transmission and receiving phases helps avoid messages collisions and unnecessary delays (only during its ON phase a node is expected to receive messages; therefore, that moment should be reserved for listening to other nodes). The complete phase-lock mechanism is depicted in Figure 3.

Despite its suitability, Contikimac imposes three major constraints:

1. Firstly, senders have to be able to predict the receiver's future ON periods. To this aim, senders must know two aspects: (i) the precise time when the receiver entered in a previous ON period, which must be obtained at run-time by means of ACKs and (ii), the periodicity of receiver's duty cycle, *i.e.*, how long its phases are. The problem with this second aspect is that the sender does not have any knowledge of this information at run-time and therefore, it must be hardcoded in nodes' program memory. This is an important disadvantage because Contikimac leaves no room for adaptive duty-cycling algorithms in which changing the duty cycle parameters is the key element.
2. The second constraint comes from the assumption that nodes can only receive messages when they are in their ON duty cycle period. This may sound obvious; however, the following example proves the existence of a gap in this reasoning: if a node (identified as A) in its sleep phase turned its radio ON to send some data (to node B), while waiting for the acknowledgement from that node (B); node A could receive a data message from another node (from node C). The acknowledgement that A would send to node C as a response could lead to a miss-synchronization between these two nodes (C would think A is in its ON phase but it just overheard the message while trying to send its own). This constraint results in discarding messages received during an OFF/sleeping period of the duty-cycle that otherwise would have been taken into account.
3. The phase-lock mechanism only works in one way. After sending the ACK, the sender knows when the receiver will enter in future ON periods, but the opposite case is not true. When roles are inverted, and the receiver becomes the sender (and similarly the sender becomes the receiver), this new sender will have to flood the receiver with preambles until it receives an ACK to synchronize with it. Therefore, we can say that this mechanism is susceptible to improvement.

To overcome these three limitations and attain maximum network performance, substantial modifications to the current Contikimac are introduced. Basically, the idea is to include in the original message a small piece of additional information referred to the duty-cycle configuration when an ACK or a data message is transmitted. This extra information can be of two different types:

1. Information regarding the remaining time until the next ON period. This allows receivers to accept and acknowledge messages received out of their ON period by simply letting the sender learn about the duty cycle configuration of the receiver. Furthermore, adding duty-cycle information in data messages provides the receivers the ability to synchronize with the senders – and not only the senders with the receivers as occurs in WiseMac or the current Contikimac implementation. This is especially important in a Smart Grid context, where information flows in both ways. Furthermore, with a minimum cost of one message exchange (and an ACK), a pair of nodes, regardless of their role (sender/receiver), can get synchronized.
2. Information about the duration of the ON/OFF periods. This information is added in order to enable adaptive duty-cycle functionalities, which have been already proved in previous studies [54] to be of great value for improving the performance of duty-cycling mechanisms. It is worth noting that if no modification of the duty-cycle settings (ON/OFF periods) has been issued, there is no need to include this second type of extra information in a message.

It is important to understand that communications in Smart Grid environments are of a very “bursty” nature.

Nodes can remain in normal operation for long period of times only sending their sensed physical data. However, when a malfunction/non-ideal condition is detected in a power asset, the activity in the WSN greatly increases in both ways—nodes would send as much information about the incident as possible, while the central node would take decisions and return orders to nodes. Under such circumstances, to achieve maximum network performance, the MAC layer must allow nodes to transmit data messages in a bursty way. To this end, when a sender has more than one message to be dispatched in a very short period of time (as it occurs, for instance, if an incident arises), the sender will transmit one after another as they are being acknowledged by the receiver (this node remains in awake period until it does not receive any more data messages). As a contribution, it is not necessary for the sender to wait for the receiver to enter in as many ON phases as messages the sender has to dispatch, thus finding a solution to the incident more quickly.

Furthermore, a scheduling algorithm has been designed and implemented to aid nodes to find the most suitable instant to transmit their messages and thus exploiting as much as possible the physical medium. Under this premise, messages from a sender are not dispatched to different nodes following a FIFO (First in, First out) approach; but instead, messages are sent in such a way as to minimize the end-to-end delay. This mechanism seeks reducing the senders' waiting times and leads to smaller power consumption and a boost in the global network performance. In order to implement these two contributions (packet bursts and non-FIFO approach), exhaustive work has been accomplished to modify diverse TinyOS components and primitives as required.

**Figure 4.**
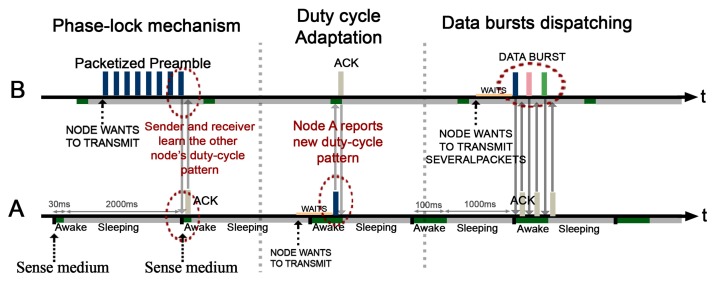
Figure of our proposed MAC layer.

The Figure 4 shows (i) the phase-lock mechanism in which both nodes get mutually synchronized; (ii) the process of adjustment/adaptation of the duty-cycle of A to, for instance, route a higher amount of messages; and a (iii) burst of data from node B.

**Figure 5.**
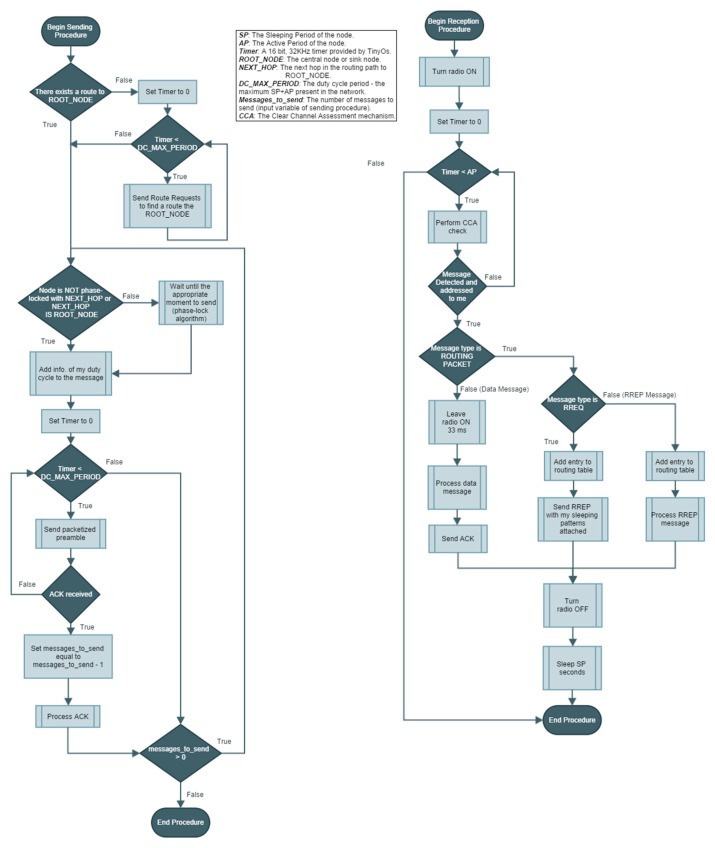
Flowchart of our proposed MAC layer. Left diagram presents the procedure for dispatching messages and right diagram the procedure for reception messages.

For further interested readers, the following flowchart (Figure 5) presents a representation of the MAC logic implemented in nodes. Two procedures are shown: the left one is in charge of handling the dispatching of messages and the right one deals with the reception of messages.

When designing an algorithm that strongly relies on a temporal accuracy—like the phase-lock synchronization mechanism—it is of great importance to take into account the hardware limitations of WSN nodes and, in particular, their clock drift. To this end, in our implementation of the phase-lock mechanism, sender nodes compensate the potential effects of clock drift in synchronized nodes by anticipating the transmission of their preamble. This “compensation time” increases as more time a sender/receiver pair spends without exchanging any message. However, in our solution, because of the acceptable clock-drift of the WSN nodes—about 30–50 ppm [58]—and the not-too-long periods without communication (maximum of 15 min as described in following sections), the clock deviation to be adjusted is, in the worst case, in the order of 50 milliseconds.

### 4.3. SENSED-SG Network Layer

In our integral WSN-based system, the SENSED-SG network layer is in charge of providing message forwarding, and in particular, accomplishing the routing tasks. Basically, this layer enables nodes out of the sink node's range to deliver their messages to this node by means of a routing protocol.

The design of a routing protocol must be based on the features of its target networks [59]; in line with this, engineering a routing protocol for a Smart Grid involves not only overcoming the challenges imposed by this environment (high electromagnetic interferences, lack of inexhaustible energy sources, *etc.*) but also exploiting some beneficial features of it, such as the absence of nodes' mobility (power assets are installed in fixed emplacements) and the robustness of the wireless network topology (although WSN nodes may fail, the probability of such event is relatively low [21]).

Bearing in mind these features, challenges and benefits, our proposed routing protocol is designed according to the following directions:

- To minimize the number of messages sent and, therefore, to reduce power consumption, instead of making use of a proactive approach, nodes will explore a route to a given destination only when required, following, to this aim, a reactive approach. Unlike proactive routing protocols, reactive solutions are mainly used in larger networks (as those used in Smart Grids) where it is not necessary to maintain an up-to-date routing table in each node with all the possible destinations (as required in proactive protocols). The construction of this routing table implies a higher routing overhead (a great amount of messages are needed to be sent) and a notable increase in memory resources. Under these premises and as starting point, we have employed the J. Kim's reactive implementation of the AODV (*Ad hoc* On-Demand Distance Vector) algorithm for the TinyOS operating system [60].
- In agreement with the effort made in reducing the number of network messages, we take advantage of the aforementioned robustness of the wireless topology: we assume that once the network topology is generated, few changes will take place in the near future. Therefore, when a node receives a route request from a neighbor, instead of re-broadcasting it (as it happens in most cases in the traditional AODV), if that node knows how to answer that request, it will directly send the reply message to its neighbor, sharply reducing the number of route request messages. This is even more significant when the routing protocol is built on top of a MAC layer specifically designed for unicast delivering. Note that route requests are messages broadcast to all possible nodes in coverage range, and therefore, these messages cannot benefit the phase-lock mechanism; resulting in a higher power consumption and a reduction in the network throughput.
- Unlike traditional routing protocols that only consider the hop count to select the best route for a message, our proposal goes beyond, including the link quality indication (LQI) as a reliable method for assessing the quality of the link and the interference level. The use of the LQI has been selected as the preferred metric to estimate the goodness of a specific link over the traditional Received Signal Strength Indicator (RSSI) in a WSN [61]. This is all the more true when a WSN operates in a hostile environment as it occurs in a Smart Grid. The SG is full of electromagnetic interferences that can produce higher values of RSSI in certain links, which in turn would trick nodes into choosing non-optimal paths. Therefore, the hop count together with the LQI will be used to estimate the best route for a message to follow.

Like most routing protocols, our approach depends on broadcasting packets to nodes in the coverage area to obtain important routing information. However, when combined with duty-cycling techniques (in which nodes are sleeping most of the time), broadcast messages are not usually heard by neighbors. To overcome this situation, messages intended to be received by all neighboring nodes, are not dispatched via the phase-lock mechanism, but instead they are continuously transmitted during a full duty cycle period, ensuring all neighbors have received the message. Since broadcasting techniques lead to higher power consumption, our routing protocol makes use of broadcast messages only when strictly necessary. Besides, it is worth mentioning that in order to not neglect relaying tasks, a specific buffering system has been implemented: if a node receives a message from a neighbor to relay it and the node under consideration is trying to dispatch its own message, this node will store the other neighbor's message until it can be sent at the appropriate moment, determined by the phase-lock mechanism. This mechanism greatly reduces the number of messages lost in more active networks.

### 4.4. SENSED-SG Upper Layers

In a traditional protocol stack, the transport layer is on top of the network level. The transport layer is responsible for providing end-to-end flow control or connection-oriented data streams among other features. However, since our system is intended to achieve maximum performance in very specific tasks (basically those required to furnish a control and monitoring system), most of the attributes designed for a general-purpose transport layer are not required and would introduce greater complexity to our solution; hence we discard popular solutions such as the User Datagram Protocol (UDP) or Transmission Control Protocol (TCP) protocols. Therefore, services to be facilitated by the transport and upper layers are comprised in a single layer on top of the network level. In there, a specific mechanism is implemented to provide each particular service (which is completely in line with an *ad*-*hoc* system).

End-to-end retransmissions and multiplexing mechanisms that are usually found in the transport layer are implemented as follows: the former is based on ACKs and timeouts to ensure the proper dispatching of data messages. When a message is sent, a timer is set. If the ACK is not received before the timeout, a retransmission is issued. This mechanism will be validated in the next section as a good and simple solution for dealing with unstable environments with high likelihood of message loss. On the other hand, the multiplexing service of the transport level is provided by incorporating an application identifier in each message, that way there can exist multiple endpoints in a single node. Different commands or report types are identified with different application IDs. The TinyOS operating system is in charge of assigning and managing these IDs.

Concerning data protection and in line with the security requirement, an encryption mechanism is implemented. The National Institute for Standards and Technology (NIST) has outlined that the preferred encryption algorithm for new deployments must be AES since other available solutions like, Triple Data Encryption Algorithm (3DES), will become insecure by the year 2030 (note that the expected lifetime of power assets is more than 50 years). The AES implementation to be used (128 bits) is fulfilled at hardware level by the radio interface CC2420 and managed via the TinyOS operating system. Encrypting messages at hardware level brings two main advantages compared to software encryption: higher speed and lower power consumption [62].

Finally, the last set of services to be implemented in the upper layer of the network level is the following one: (i) the generation of orders for sensing physical parameters; (ii) the processing of this information and (iii) the communication management. In this regard, it is worth mentioning that WSN devices are programmed to periodically acquire physical data, process it and determine the current state of the power assets they are monitoring. If a malfunction or a non-ideal condition/trend is detected (a specific threshold is surpassed or a certain condition is met) those readings must be considered as alarms and get them delivered to the central node to register the situation and, if possible, correct the deviation/problem. Furthermore, less frequently, sensor readings are collected and all the acquired information is sent to the sink node once it has been processed (this happens regardless of the obtained values). This two-way dispatching process allows the central node to keep records of power assets' states but not at the expense of drastically increasing the power consumption of nodes—caused by frequent and non-relevant data sending. The frequency of data sensing or information reporting (sending messages to the sink node) can be adjusted at run time to allow (i) critical power assets to report data more often (increasing the frequency) and (ii) less important assets to help extend the lifespan of their WSN devices (reducing the frequency). This process of data sensing/reporting frequency adjustment is achieved by central-node commands. The central node may instruct deployed nodes to change their sensing/reporting frequencies or even order them to send fresher sensors' readings in case a clearer picture of the overall system is needed. Note that defining the default frequencies for both processes (sensing and reporting) is a complex task. Shortening any of these periods will result in a reduction of nodes' lifespan whereas increasing it will jeopardize the ability of the system to react to unexpected situations (longer sensing/reporting periods implies a smaller number of reports about the state of the SG).

In view of the proposed solution and the difficulty of adjusting certain parameters (reporting or sensing frequency and duty-cycling parameters among others) the next section provides a set of simulations and analyses that help choose the most suitable values. However, it should be noted that there is no silver bullet for every possible scenario and there always exists a trade-off when choosing specific values; therefore the final decision will depend on many factors that will be extensively commented in the next section.

## 5. Analysis of the Solution and Simulation

### 5.1. Analytical and Validation Study

A theoretical study using analytical expressions and simulation tools allow us to conduct experiments using a system model before deploying the real devices on the field. This study results in a better understanding of the *design parameters*, determining their appropriate ranges and enhancing the use of available resources. The goal is to achieve a suitable performance of SENSED-SG for its subsequent real-operation in a Smart Grid. Consequently, in order to assess diverse figures of merit, we have, on the one hand, derived their most appropriate mathematical equations that can be applied to a Smart Grid and, on the other hand, implemented our proposal in two simulation scenarios. Furthermore, using these figures of merit as the metrics of interest, we have compared our proposal with a ZigBee approach to demonstrate its feasibility. The chosen metrics are the following ones:

- **Packet Reception Rate (PRR)**: The ratio between the number of received messages and the total amount of messages sent (for an arbitrary pair of sender/receiver nodes). It gives an idea of how well the network performs in term of losses.
- **End-to-end delay**: The time elapsed between when a message is generated and it reaches the receiver and gets processed. This metric is especially relevant for scenarios characterized by hard real-time constraints where the “freshness” of the information is crucial.
- **Energy Consumption**: The total energy consumed by nodes after a given period of time. Since nodes are not equipped with inexhaustible power supplies, this metric is of tremendous importance to extend nodes' lifetimes, and therefore, the network lifetime of the WSN deployed.

Although there is a notable amount of *design parameters* capable of affecting these three metrics in some way, this work is focused on the effects of varying (i) the awake and sleep periods of the duty cycle and (ii) the sensing and reporting frequencies. The duty cycle configuration has a direct impact on all three metrics whereas the reporting and sensing frequencies basically influences the total energy consumed per node. The reason for choosing these parameters instead of any others is twofold: firstly, their higher impact on the metrics compared to others (e.g., the CSMA backoff or the periodicity of route discarding in AODV). And secondly and more important, all of the chosen ones can be adjusted and tweaked at real-time to adapt the network to the dynamics of the Smart Grid.

To this end, two approaches will be considered: (i) a mathematical characterization that quantifies the effects of the design parameters on the figures of merit selected. This is done to obtain the analytical expressions that help to calculate the network behavior in future deployments. And (ii) a set of simulations of the network for representative environments is also carried out to *verify and validate* those *numerical expressions*. In this respect, one of the inherent problems that arises in computer-oriented network simulators is the lack of fine-grained simulation of the nodes behavior at hardware level (in particular, the radio interface and microcontroller specific behaviors). These limitations are not generally taken into account by widespread simulators and play an important role in the global behavior of the system. The problem is aggravated when these simulators ignore a specific node's hardware constraints like memory or computational processing limitations. To relieve this problem, instead of traditional computer-oriented simulators (like ns-2 or Omnet++), embedded-systems-oriented simulators (such as Avrora and TOSSIM) will be employed.

Regarding the simulation frameworks, Avrora [63] is a cycle-accurate instruction-level sensor network simulator capable of measuring detailed time-critical phenomena like the power consumption of each node operating in the WSN. This is the main reason why it is specifically used for providing an in-depth knowledge of WSN devices' consumption profiles. Along with Avrora, the TOSSIM simulator is employed in our work to emulate different propagation environments and noise patterns. The choice of TOSSIM is twofold, firstly it simulates the same code that is uploaded into WSN devices, easing and empowering the debugging process; and lastly it enables the definition of propagation and noise parameters, allowing us to accurately study very specific environments.

**Figure 6.**
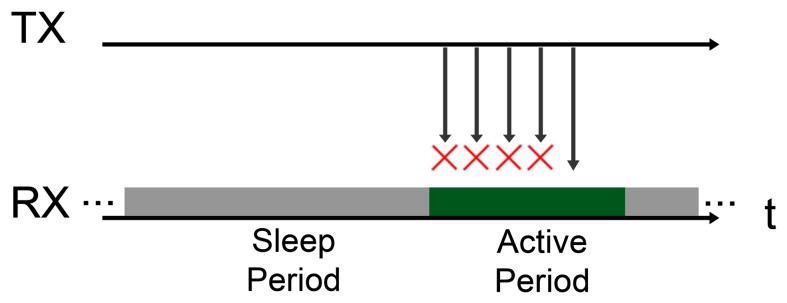
Figure of our proposed MAC layer.

As explained in the previous section, every node switches from the sleep state to the awake state and then returns to sleep periodically. The duration of both states is denoted as **AP** for the awake period, and **SP** for the sleep period. A sender will strive to send its messages in the forthcoming receiver's AP. One of the consequences of the duty cycle is that the shorter the receiver's AP, the fewer attempts the sender will have to get its messages delivered. This situation is depicted in Figure 6 where the red crosses represent message losses due to interferences or collisions. The first relation between design parameters and metrics can therefore be identified: reducing the Active Period will also decrease the number of potential attempts that the sender does for delivering its messages. Consequently, the likelihood of message loss will increase.

Defining *P(L)* as the probability of message loss and *N* as the number of attempts, the PRR can be expressed by Equation (1):

(1) $$\text{PPR}=1-P{\left(L\right)}^{N}$$

**Figure 7.**
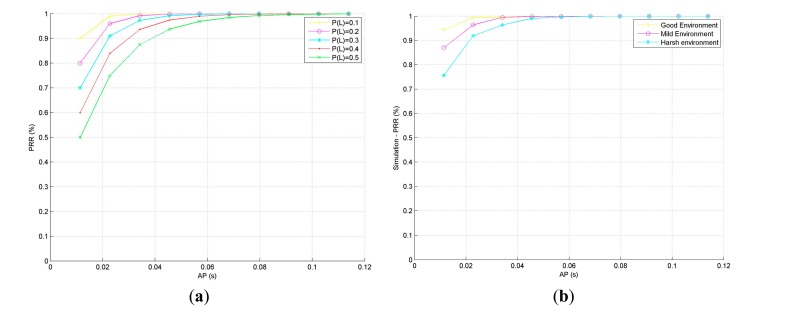
(**a**) Analytical results of PRR *vs*. AP for different values of P(L); (**b**) TOSSIM Simulation results of PRR *vs*. AP for different environments.

The Figure 7a illustrates the value of PRR for different scenarios. As expected, in this figure, we can observe how the PRR increases as the AP grows. This phenomenon is also confirmed by means of TOSSIM simulations (Figure 7b). To this aim the following simulation configuration is set:

- **Topology:** Two nodes in coverage range.
- **Propagation environment:** These two nodes are deployed in three different scenarios. The first one is characterized by very harsh radio propagation conditions (Harsh environment—average probability of packet loss: 0.25). The second environment is defined by its moderately hard propagation conditions (Mild Environment—average likelihood of packet loss: 0.13). And finally the last scenario represents a favorable environment where the average likelihood of packet loss is around 0.05 (Good Environment). These probabilities of packet lost have been selected as a result of a previous study conducted to characterize different SG propagation environments and are implemented in the simulator by tuning different design parameters of the log-distance path model (path loss exponent, shadowing standard deviation, power decay, *etc.*). This model has been widely substantiated and validated in multiple WSN-based approaches applied to SG [64,65] and therefore, it has been selected as the propagation model for every simulation carried out in this work.
- **Message size:** The length of the message is 15 bytes.

Due to the CSMA/CA mechanism (total transmission time, ACK waiting time and backoff among others) and hardware limitations (clock frequency of the transceiver and processing unit) nodes can carry out one attempt of transmission each 11 milliseconds; therefore, the Active Period grows in increments of 11 ms to minimize the time the radio is ON—in both figures AP varies from 11 ms to 110 ms (maximum value computed that corresponds to 10 attempts of retransmission).

It can be observed that extending the AP from 11 ms to 33 ms (from 1 attempt to 6), the PRR increases up to a 49% in very harsh scenarios. Therefore, more attempts mean a higher probability that messages reach the destination. This is especially important in the Smart Grid, where nodes are expected to be deployed in very noisy and unsteady environments. Then, in the interest of enhancing the network's reliability, the AP should be extended until guaranteeing a significant value of PRR.

The node power consumption during *the duty cycle mechanism* (energy employed by the radio interface during sleep and periodic listening) is easily calculated if power-consuming profiles of both, the AP and SP are identified. Defining *T_AP_* as the time the node spends in awake mode, *T_SP_* as the time the node spends in sleep mode, *I_AP_* and *I_SP_* as the currents drawn in awake and sleep mode respectively and V as the voltage supplied by the batteries, the energy consumed is expressed by Equation (2):

(2) $$E\left(J\right)=\left(T_{AP}*I_{AP}+T_{SP}*I_{SP}\right)*V$$

**Figure 8.**
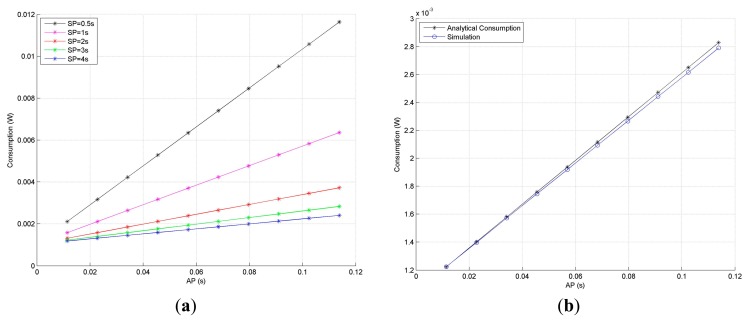
(**a**) Consumption *vs.* AP for different values of SP—Mathematically obtained. (**b**) Consumption *vs.* AP for a fixed value of SP = 3s—Comparison of analytical and simulation results.

Unlike the PRR, the power consumption is sharply affected by the duration of AP and SP. As depicted in Figure 8a, the greater the AP, the more energy is consumed; and hence, the first tradeoff is found: to increase the PRR, the AP must be enlarged; however, doing so would increase the power consumption and reduce nodes' lifetime. Figure 8b illustrates the correctness of the Equation (2) comparing the calculated values with the simulation results obtained by means of Avrora. As already commented, in these simulations the precise consumption profiles of the microcontroller and the radio transceiver are taken into consideration.

**Figure 9.**
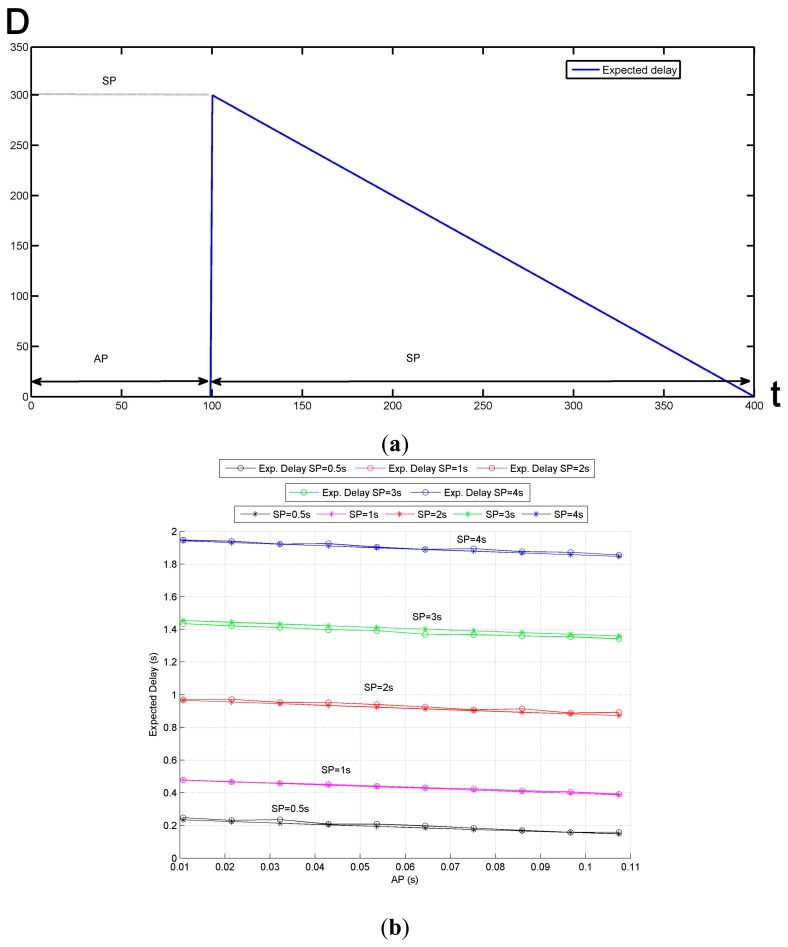
(**a**) Representation of the expected delay and its dependence on AP and SP; (**b**) Mathematical and simulated delay values for a given pair of nodes.

As regards to the delay, the analytical expression is derived in Equation (3) obtaining the expected delay (*E[D]*). When node A wants to transmit a packet to node B (next hop in the route to root node), the transmitter (A) must wait a certain amount of time *D* until the receiver (B) enters in its Active Period (*AP*) (this is due to the phase-lock mechanism). Therefore, *D* ranges from 0 s (A wants to transmit at the very moment B is in its AP) to *SP* seconds (A wants to transmit at the very moment B enters in its sleeping period—*SP*). The following figure (Figure 9a) models the waiting time *D* and its dependence on *SP* and *AP*. The abscissa axis represents the precise moment A wants to transmit its packet and the ordinate axis represents the corresponding waiting time *D*. To obtain the expected delay (equation denoted as 3) it is necessary to average the values of *D* over the entire AP+SP period, *i.e.*, find the average value of such geometrical figure:

(3) $$E\left[D\right]=\frac{1}{T}\int_{-\infty }^{\infty }f\left(x\right)dx=\frac{1}{AP+SP}(\int_{-\infty }^{AP}0+\int_{AP}^{AP+SP}\left(-x+AP+SP\right)dx)=\frac{\left(AP+SP\right)}{2}+\frac{AP^{2}}{2(AP+SP)}-AP$$

The analytical values in Figure 9b ignore the effects on the delay of different phenomena such as processing time or radio propagation effect. However, as can be observed in the comparison of mathematical and simulated values plotted in Figure 9b, these omitted extra delays do not have a meaningful impact on the overall delay.

Finally, if the AP is small (as it usually occurs), the expected delay can be approximated to a half of the SP—as observed in Figure 9b. As already mentioned, in the Smart Grid the end-to-end delay should not exceed fifteen seconds, therefore for a typical communication network of up to 10 hops, the SP should always be lower than 3 s (a SP of duration 3 s generates a delay of about 1.5 s between a pair of nodes which, multiplied by the 10 hops, results in 15 s).

It is worth mentioning that the analytical model here proposed is based on a series of assumptions that simplifies the associated formulae. Such assumptions allowed us to propose a model that reflects the interaction between only two nodes—a transmitter and a receiver—and therefore, unlike many other works ([66,67]) it can only be applied to SENSED-SG. Such assumptions can be described as follows: (i) nodes send messages at a relatively slow rate (unsaturated traffic regime); (ii) message collisions are very unlikely to occur (the backoff of the CSMA algorithm is not taken into consideration) and (iii) the transmission and acknowledgement of packets take place in a very short period of time (therefore the *transmitting* and *waiting* states are not modeled).

### 5.2. Simulation Scenario

To validate the effectiveness of the proposed network and to compare it with other well-accepted WSN solutions (e.g., ZigBee), a set of demonstrative simulations is conducted. For this purpose, it is mandatory to determine the AP and SP durations of the duty cycle. In order to reduce the number of packet losses in the SG environment, and following the same reasoning of the previous section, the AP is set to 33 ms (up to three sending attempts can be carried out by the sender). Besides, the SP is set to 1 s to reduce the delay and enable the deployment of bigger networks (up to 30 hops). This configuration will not jeopardize the long-standing feature of the network as shown in the following paragraphs.

Various simulations are carried out to evaluate the response of the proposed network to each of the propagation environments introduced in the PRR analysis (Section 5.1). Asides from using the aforementioned log-distance path loss model, the TOSSIM simulator exploits the benefits of a very robust environment-aware noise model named Closest-fit Pattern Matching (CPM) [64]. CPM has proven to increase the packet simulation fidelity by up to a factor of 5 [68] in comparison with other simulation approaches (such as those used in ns-2 [69] or EmStar [70]). Furthermore, CPM noise model clearly achieves its best performance when fed with noise traces taken from the real environment to be simulated (this helps to characterize the different interfering phenomena of specific surroundings). Therefore, to enhance the accuracy of the simulation, a noise trace obtained in a power environment is included in our simulations. This way, unlike many other works ([8,33,71]), both propagation environments and noise patterns are considered in our simulations—this is especially important in the Smart Grid arena where the electromagnetic noise has as much impact on the final performance as the radio propagation environment itself. The TOSSIM simulator has been fed with the values of Table 1 so as to simulate the three desired scenarios.

The *Path loss exponent* models the rate at which signal decays when it propagates in a specific environment. The *Shadowing standard deviation* represents the randomness of the received signal due to diverse phenomena (objects in the line of sight, multipath, *etc.*). *Power decay*, models the power (in dBs) presence at 1 m distance from the transmitter. Finally *Noise floor* represents the received power of other noise sources and other unwanted signals (expressed in dBs). Such values have been obtained by carefully reviewing academic literature ([9,65,68,72]) together with a deep analysis of the simulator reference tutorial found in [73].

To the best of our knowledge, there is no WSN simulation for the Smart Grid that simultaneously integrates specific network-layer protocols, MAC and low-power listening mechanisms (authors tend to analyze separately each of the aspects of the WSN, neglecting the effects that one layer may cause to the other). Since SENSED-SG implements a protocol stack composed of the application, the network, the MAC and the physical layers; our simulations jointly evaluate all these elements. Furthermore, constant data-generating patterns are usually assumed in specialized literature [33,35]. This fact causes that the very bursty nature of a WSN operating in a Smart Grid is neglected, obtaining results that greatly differ from those found in real deployments. To address this concern, in our simulations, bursts of data are randomly generated to emulate the presence of non-desirable conditions in power assets (which usually leads to an increase in data sending).

**Figure 10.**
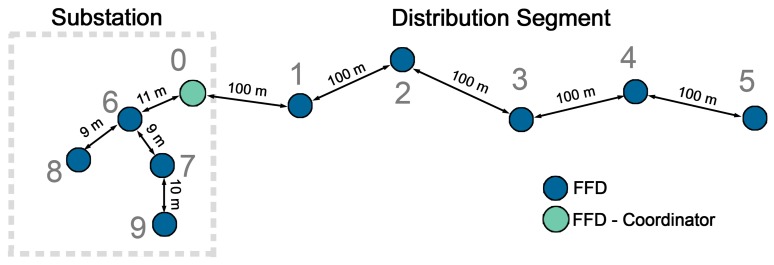
Simulated topology.

The topology under simulation is shown in Figure 10. In the interest of providing a general picture of the network performance, two potential node distributions are taken into account in the same topology: nodes deployed in the framework of an outdoor substation (cluster-tree topology and smaller separation distances between nodes) and nodes placed in electric towers along the distribution segment of the power grid (following a line topology). Nodes 1–5 are equipped with vibration sensors (which can be used to monitor for excess vibration in the electric towers and aid in determining their structural integrity) and temperature sensors to check the temperature variations in the conductors (identified as the major cause of sagging). On the contrary, nodes 6–9, deployed within the substation, (and placed in transformers, circuit breakers, feeder bays, *etc.*) include temperature and acoustic sensors to detect potential partial discharges and monitor the operating temperature. To this aim, the MTS310 environmental sensor board [74] (fitted with a temperature sensor, an acoustic sensor and a dual-axis accelerometer) is simulated. Physical data (sensors readings) are acquired every 5 min (*sensing frequency*), processed, and sent if the asset is failing in simulated data bursts. Besides, every 15 min sensor readings are obligatorily sent to the central node regardless their value to have an updated record of the status of assets (*reporting frequency*). The central node processes all of this information and, if a faulty functioning is detected in any power asset, simulated commands are sent back to nodes to correct these eventualities.

The results achieved with our approach are compared to a non-beacon ZigBee network where nodes 5, 8 and 9 (RFD) use duty-cycling techniques whereas the rest of them (FFD) must remain awake to route RFDs' messages. The reason why a non-beacon network has been selected instead of a beacon-enabled network is twofold: firstly, a beacon-enabled network is not a flexible and scalable solution that could be deployed in a Smart Grid environment, and therefore, *a priori*, it cannot be used in real scenarios. Lastly, although there exist two approaches for implementing a beacon-enabled network in a cluster-tree topology, namely *time division approach* (TD) and *beacon-only period approach* (BOP), both of them exhibit many drawbacks. The TD implementation prevents the use of broadcast messages between FFDs, which negatively affects the operation principle of the AODV protocol and excludes the creation of multi-hop routing paths. On the contrary, in the BOP approach, beacon broadcasters (FFDs) are forced to be deployed at precise locations to ensure global network synchronization [75]. These locations might have no power asset to control.

Concerning the power consumption calculations, the following two considerations are taken into account. Firstly, vibration and acoustic readings take 3 s to be completed (because of sensor stabilization times) whereas the temperature can be read in just 25 ms. Secondly, the current draw (according to their corresponding datasheets and [76]) is as follows: 9.5 mA for the accelerometer and the acoustic sensor, and 7.5mA for the temperature sensor.

### 5.3. Simulation Results and Comparison with ZigBee

**Figure 11.**
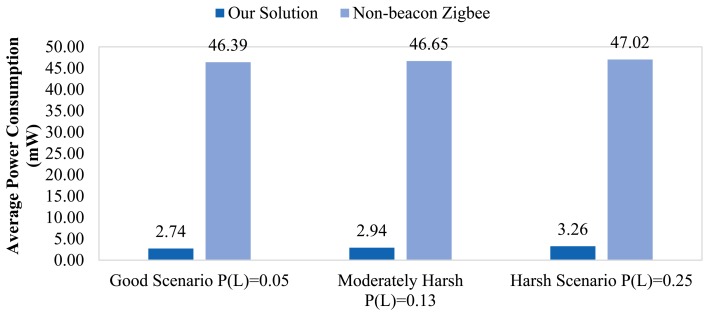
Power Consumption of SENSED-SG *vs.* a non-beacon ZigBee-based network for three different scenarios.

Figure 11 illustrates the average node power consumption (PC) of our proposed solution *vs.* a non-beacon ZigBee-based network for three different cases: good, moderately harsh and harsh scenarios respectively. The higher power consumption of the non-beacon ZigBee solution is mainly caused by the absence of duty-cycling mechanisms in full function devices, which, in turn, leads to faster FFDs' batteries depletion. When routing nodes (FFDs) exhaust their batteries, some WSN devices in the network may become partially isolated, being impossible for these nodes to dispatch their messages successfully to their destination. This is why the *network lifetime* (as a concept) is defined as the time elapsed between the beginning of the network operation and when the first node depletes its battery. Under this consideration, the expected network lifetime is shown in Table 2.

As written in Table 2, our solution lasts from 16 to 20 times longer than a non-beacon ZigBee-based network. Even in the harshest scenario, our proposed network is expected to be operating for more than 16 months. Thereby, SENSED-SG is more appropriate for deployments in remote locations or hard-to-reach areas. This long-lasting feature can be further enhanced by reducing the sensing or reporting period; for instance, if physical data is acquired every 15 min (instead of every 5 min), the network will operate for more than 24 months under good propagation conditions. On the contrary, the experiments reveal that a non-beacon ZigBee network is not suitable for deployments where inexhaustible power sources are not available, discarding it as a potential solution for almost every segment of the Smart Grid (such as the Generation and the T&D segments).

**Figure 12.**
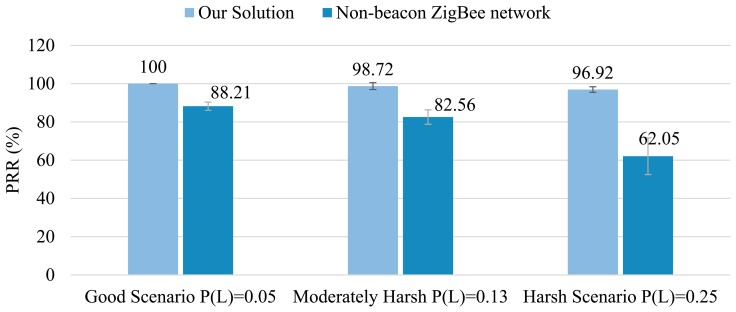
Packet reception ratio of SENSED-SG *vs.* non-beacon ZigBee for three different scenarios.

As regards to the packet delivery ratio, Figure 12 shows the average PRR and its standard deviation with a 95% confidence interval, obtained from 15 simulations carried out with different seeds. Results evidence that SENSED-SG performs adequately even under harsh environments. This is achieved by combining retransmission algorithms with appropriate modulation techniques and by carefully selecting the best MAC settings (e.g., the active/sleep period, retransmission times, *etc.*). It is worth highlighting the obtained robustness of our solution to harsh propagation conditions, decreasing in just 3.08% the PRR if the probability of packet loss increases from 5% to 25%—as opposed to a decrease of 26.16% in the PRR in non-beacon ZigBee networks.

Since routing nodes (FFDs) remain in awake mode all the time in non-beacon ZigBee-based networks (and, therefore, these nodes do not have to wait an AP for sending messages), the delay obtained is lower with this approach than with the SENSED-SG. However, in our proposal, the attained average network delay is kept below 1.2 s in every scenario, with a maximum peak of 2.3 s reached in the node No. 5 (the farthest WSN device from the sink-node). These results satisfy the delay requirement imposed to WSN operating in Smart Grids, which must be lower than 15 s.

To summarize, we would like to highlight and further comment the simulation results:

- The network lifetime obtained with SENSED-SG in each scenario (good, mild and harsh) is: 20.85, 18.9 and 16.8 times higher respectively than the one obtained with a ZigBee-based network.
- The PRRs achieved with SENSED-SG in each scenario (good, mild and harsh) are 13.4%, 19.6% and 56.2% higher than the ones obtained in a ZigBee-based network.
- The delay, as a result of the extension of the network lifetime, is in SENSED-SG, on average 1.1 s higher.

In light of the above simulation results, the feasibility of our proposal for furnishing a reliable and cost-effective asset-managing system for the Smart Grid has been widely demonstrated. These simulations, despite having taken into account a large amount of potential aspects (such as different propagation environments, various interfering phenomena, node's hardware constraints, diverse topologies, *etc.*), inevitably still represent a simplification of the real world. To address this weakness, we complete our work with a series of test-beds experimentation presented in the next section. They are carried out to prove the suitability of the solution here proposed from a higher perspective and, to further validate the results obtained by the analysis and the simulation frameworks (Sections 5.1 and 5.3).

## 6. Test-Beds

From a communication point of view, the Smart Grid is characterized by a very complex and unsteady nature. Simulators deal with this concern by using realistic-enough propagation models and (in the case of TOSSIM along with its CPM) by incorporating some key features of the real environment, such as noise pattern. However, some phenomena are difficult to reproduce and are usually simplified or even neglected. For instance, shadowing deviation is simplified by a model based on a log-normal expression whereas the adjacent-channel interference effect is simply omitted. Therefore, the only way to ensure that those phenomena do not have a major impact on the network performance is by running a set of well-controlled test-beds which will be used, in addition, to validate the results obtained by both, analysis and computer simulation.

**Figure 13.**
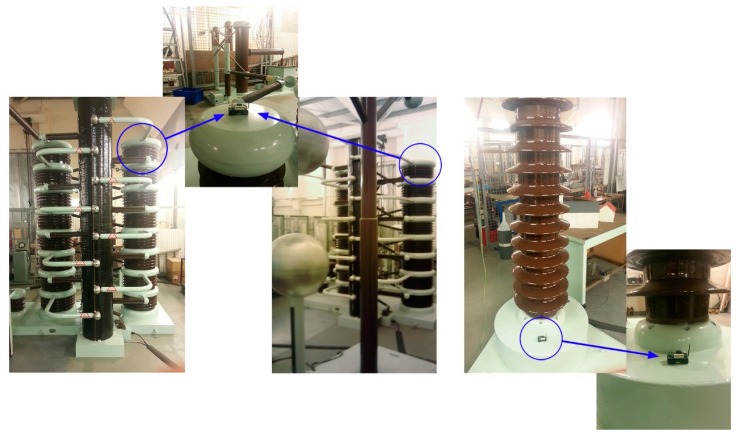
Experiment setting in Heriot-Watt High-Voltage Lab.

As in the simulation study, two representative scenarios are considered in the test-beds: an electrical substation (indoor) and the SG distribution segment (outdoor). Since in an electrical substation too many elements can interfere and thus affect the results, nodes are deployed in a controlled environment: the High Voltage AC/DC laboratory of the Heriot-Watt University (Edinburgh Campus)—depicted in Figure 13. There, the topology in Figure 10 is built inside a Faraday cage where the equipment (transformers and circuit breakers among others) is energized to simulate the normal functioning of power assets and the resulting electromagnetic noise. In particular, nodes 0, 6, 7, 8 and 9 are deployed in the laboratory, placing WSN nodes close to power assets and dispatching the same amount of information as in the simulation phase as a proof of concept. As in simulations, the sensing and reporting periods are left untouched, being 5 and 15 min respectively. The packet size is fixed to 15 bytes and the transmission power of the devices is set to its default value as well. Regarding the second test-bed, the same settings as in the previous experiment are employed (same values for the sensing and reporting frequencies, packet size and transmission power) and nodes 1, 2, 3, 4 and 5 of the topology illustrated in Figure 10 are deployed in a T&D segment as a demonstration of the proper operation of our proposal in an outdoor environment. The distance between two consecutive nodes is approximately 100 m (distance between transmission poles) and the only interfering phenomenon is the electromagnetic noise generated by the distribution segment itself. Nodes are deployed on the electrical towers and, as in the former test-bed, their readings periodically requested.

The PRR and the delay are continuously monitored all along the experiments to detect any deviation from the simulation results. On the other hand, power consumption depends on the protocol stack and the use of hardware resources (mainly the microcontroller and the radio interface), which are thoroughly evaluated by the Avrora simulator. Avrora simulations have been proved to be of remarkable accuracy by many works [63,77]; however, being one of the key points of the proposed system is its long-lasting feature, we have considered that a validation of the power consumption simulation results is of paramount importance. To this aim, the power consumption of the seventh node has been examined for an entire week. This measurement has been carried out by periodically sampling the current drawn by the WSN node from a power source. After a week we obtained a value that is 99% equal to the one predicted by the simulations—being the actual power consumption slightly lower than expected. Therefore, we consider that, in general, simulated power consumption matches the reality and therefore, the solution here proposed is, as aforementioned, of long-lasting nature.

**Figure 14.**
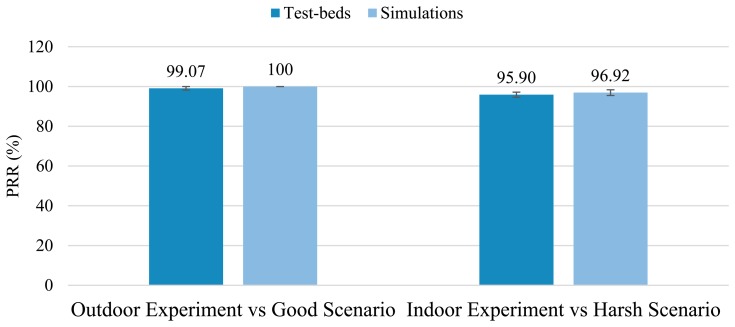
PRR—Test-bed *vs.* simulation results.

**Figure 15.**
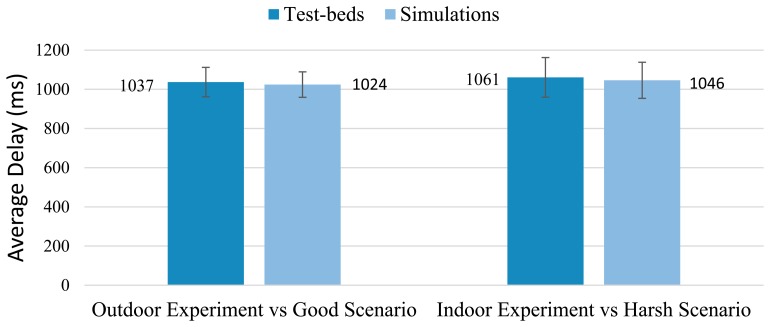
Average Delay—Test-bed *vs.* simulation results.

Regarding the PRR and Delay values, the results show the average obtained after 10 repetitions of a one-hour run (for each indoor/outdoor scenario) together with their corresponding standard deviation (95% confidence interval, Batch Mean Method). They are represented in Figures 14 and 15.

Figures 14 and 15 show how the test-bed results are quite similar to those obtained in the simulations (presented in the Section 5). The outdoor environment, although covering longer distances between nodes, is ultimately characterized by a less hostile environment (in terms of electromagnetic noise), line-of-sight communications and bigger first Fresnel zone clearance (which has a positive effect on radio propagation and reduces the signal cancelation). Thus, it is easy to understand that this experiment shows greater similarities to the *Good Scenario* simulation results. On the other hand, the substation indoor environment is affected by hard electromagnetic interferences, the absence of line-of-sight (in almost every pair of nodes) and the presence of many obstacles in the first Fresnel zone; finding the results obtained matching-enough to those obtained in the simulations under the *Harsh Environment* label.

It is worth mentioning that in order to also validate the results obtained in the simulation of the ZigBee-based network, we decided to carry out the outdoor and power-consumption experiments again, but this time, programming devices to form a ZigBee network. For the case of the power consumption analysis, we let them run another week, while testing the consumption of the seventh node: the results were 98.15% equal to the value obtained with the simulations—slightly higher in the test-bed than expected. The PRR obtained in such outdoor experiment was 89.15% (eighty-nine percent of the messages sent by nodes were received by the sink node); differing in just 1.13% from the simulation.

These experiments clearly validate the results obtained in both, the simulation and the mathematical expressions and therefore, demonstrate the suitability of our SENSED-SG approach from a global perspective to provide a reliable control and monitoring means for the Smart Grid.

## 7. Conclusions and Future Work

The Smart Grid is a challenging field full of opportunities and benefits that only very recently has started to be completely exploited. However, it still lacks *ad*-*hoc* control and sensing wireless systems capable of facing the very specific challenges and requirements that the SGs impose. In this line we have conceived, carefully justified its design, and implemented a comprehensive *ad*-*hoc* WSN solution, denoted as SENSED-SG. It benefits from the collaborative, low-cost and extremely low power consumption nature of WSNs to control and manage power assets. To this end, our solution makes use of specific implementation of the MAC, network and upper layers. Mathematical expressions have been proposed to characterize the main metrics of interest of the SENSED-SG network, and they have been further used to tweak and improve the performance of such network. The analysis has been validated by means of an exhaustive set of representative simulations. The mathematical model was also useful to predict how different figures of merit vary as different design parameters are changed, which yields trade-off values for the main system parameters.

As a way to compare our solution to other widely deployed commercial alternatives, we have also simulated a ZigBee-based network. Results reveal the good performance achieved by our proposal, especially in terms of lifetime and PRR: almost 21 months of lifespan vs. the 30 days of the ZigBee solution, and a PRR of nearly 100% in the worst case scenario in comparison with the 62% of the ZigBee-based network. Finally a test-bed has been also carried out. The indoor scenario has been replicated in a well-controlled environment: the High Voltage AC/DC laboratory of the Heriot-Watt University. There, different propagation phenomena have been considered (electromagnetic noise, scattering, shadowing, *etc.*) and results confirmed the suitability of SENSED-SG for controlling and managing different types of power assets. Conversely, an outdoor-environment test-bed has been also set in an open field where nodes were placed on electric towers to evaluate the performance of our proposal in the T&D segment of the SG. Again, the results demonstrated the reliable and robust nature of the proposed system, satisfying all the imposed requirements.

WSNs are expected to play an even more important role in the future Smart Grid, being a ubiquitous sensing solution and running unattended for longer time spans. In this sense, finer energy-harvesting techniques (especially those related to electromagnetic fields) are envisioned to empower WSN devices, providing them with the required energy to perform more complex tasks and allowing them to individually and progressively take low-impact decisions releasing the load of central nodes. Thus, the burden of the decision-making process is expected to be distributed among nodes, reducing the network overload and increasing the efficiency of the WSN. In view of the foregoing, it is our intention to work on these new energy-harvesting techniques and to incorporate new type of sensors to provide more meaningful information to the decision-making process and enable a real decentralized monitoring system. The application of cloud computing and virtualization techniques to the monitoring networks must be also explored as a means of reducing energy consumption and improving the intelligent management of large-scale WSN for the Smart Grid.

## Figures and Tables

**Table 1.** Network expected lifetime.

	**Good Scenario**	**Mild Scenario**	**Harsh Scenario**
Path loss exponent	2.15	2.31	2.93
Shadowing std. Deviation	2.45	3.15	3.33
Power decay	33.0	54.0	54.0
Noise floor	−97.0	−93.0	−85.0

**Table 2.** Network expected lifetime.

**Network Lifetime**	**SENSED-SG**	**Non-Beacon ZigBee Network**
Good Propagation Conditions	20.75 months	30.28 days
Moderately Harsh Propagation Conditions	18.71 months	30.13 days
Harsh Propagation Conditions	16.54 months	29.91 days
